# Casein Kinase 1 Epsilon Expression Predicts Poorer Prognosis in Low T-Stage Oral Cancer Patients

**DOI:** 10.3390/ijms15022876

**Published:** 2014-02-19

**Authors:** Shu-Hui Lin, Yueh-Min Lin, Chung-Min Yeh, Chih-Jung Chen, Mei-Wen Chen, Hsiao-Fang Hung, Kun-Tu Yeh, Shun-Fa Yang

**Affiliations:** 1 Institute of Medicine, Chung Shan Medical University, Taichung 402, Taiwan; E-Mail: 74630@cch.org.tw (S.-H.L.); 2 Department of Surgical Pathology, Changhua Christian Hospital, Changhua 500, Taiwan; E-Mails: 93668@cch.org.tw (Y.-M.L.); 28935@cch.org.tw (C.-M.Y.); 132540@cch.org.tw (C.-J.C.); 3 Department of Medical Technology, Jen-Teh Junior College of Medicine, Nursing and Management, Miaoli 356, Taiwan; E-Mail: tomhong3@gmail.com (H.-F.H.); 4 School of Medicine, Chung Shan Medical University, Taichung 402, Taiwan; 5 Tumor Center, Changhua Christian Hospital, Changhua 500, Taiwan; E-Mail: 135442@cch.org.tw (M.-W.C.); 6 Department of Information Management, Chien kuo Technology University, Changhua 500, Taiwan; 7 Department of Medical Research, Chung Shan Medical University Hospital, Taichung 402, Taiwan

**Keywords:** casein kinase 1 epsilon (CK1ɛ), oral cancer, overall survival

## Abstract

Casein kinase 1 is a group of ubiquitous serine/threonine kinases that are involved in normal cellular functions and several pathological conditions, such as DNA repair, cell cycle progression, cytokinesis, differentiation, and apoptosis. Recent studies have indicated that casein kinase 1-epsilon (CK1ɛ) and casein kinase 1-delta (CK1δ) expression has a role in human cancers. We investigated the associations between CK1ɛ and CK1δ expression and the clinical parameters of oral cancer using immunohistochemical study methods on oral squamous cell carcinoma specimens. The results of our immunohistochemical analysis showed that the loss of CK1ɛ expression was greatly associated with a poor four-year survival rate in oral cancer patients (*p* = 0.002). A Kaplan-Meier analysis showed that patients who had a loss of CK1ɛ expression had a considerably poorer overall survival rate than patients who had positive CK1ɛ expressions (*p* = 0.022). A univariate analysis revealed that patients who had a loss of CK1ɛ expression had considerably poorer overall survival (OS) than patients who had positive expression (*p* = 0.024, hazard ratio (*HR*) = 1.7). In conclusion, our data indicated that the loss of cytoplasmic CK1ɛ expression is greatly associated with poor survival and might be an adverse survival factor.

## 1. Introduction

Oral squamous cell carcinoma (OSCC) is the fifth most common malignancy in Taiwan; it is also the leading cause of cancer-related deaths worldwide [1,2]. Data that the Bureau of Health Promotion, Department of Health collected in 2012 indicated that patients with oral cancer are more susceptible to develop a secondary cancer than most people [1]. Moreover, patients with oral cancer have a 2.4-fold risk to get secondary cancer than patients with non-oral cancers. Men with oral cancer have a 13.6-fold recurrence rate, which is higher than that of other men with non-oral cancers. Betel chewing, cigarette smoking, and alcohol consumption have been identified as major etiological factors in oral cancer [2,3]. OSCC represents more than 90% of all oral carcinomas. Surgery, radiation therapy and chemotherapy remain the main treatments for OSCC patients, but the therapeutic effects remain unsatisfactory, and the treatment has a poor 5-year-survival rate [4]. The unequivocal prognostic and/or predictive significance of the molecular markers of oral cancer are still not established [5–10]. A prognostic marker could be used as a therapeutic target to develop effective treatment guidelines for oral cancer.

In recent years, more and more protein kinases and phosphatases have become targets for drug development, and recently, interest in specifically targeting members of the casein kinase 1 (CK1) family has increased [11]. CK1 is one of the family of serine/threonine protein kinases. CK1 kinases exist in at least seven isoforms (α, β, γ1–3, δ, and ɛ) in mammals [12,13] and CK1 kinases phosphorylate various substrates to play vital roles in diverse physiological processes such as DNA repair, cell cycle progression, cytokinesis, differentiation, and apoptosis [12–14]. CK1-epsilon (CK1ɛ) is a protein product of the *CSNK1E* gene and has been shown to be essential in regulating cell division and tumor growth in human pancreatic adenocarcinoma and salivary gland cancer by phosphorylating key proteins in the Wnt signaling pathway [15–18]. Changes in CK1ɛ expression and activity as well as the occurrence of mutations within the coding region of CK1ɛ have been reported in various cancers including mammary ductal carcinoma, ovary cancer, and breast cancer [19,20]. Moreover, CK1 epsilon molecules could be used as potential therapeutic targets in the treatment of digestive cancers [20]. However, the potential role of expression levels as a prognostic biomarker in oral cancer has not been investigated. In this study, we examined the expression of CK1ɛ in a large collection of oral cancer tissue samples to assess if CK1ɛ might serve as a predictor of outcomes. We also attempted to assess associations between CK1ɛ expression and clinicopathological parameters of oral cancer patients and the relationship to outcomes.

## 2. Results and Discussion

### 2.1. Patient Characteristics

A total of 195 patients, including 159 men and 36 women were analyzed in this retrospective study. The patients’ characteristics, including the patients’ sex, age, cancer stage, lymph node status, histological grade, tumor status, smoking habits, betel nut chewing habits, and overall survival are listed in Table 1. The mean age of the 195 patients was 55.9 years (a range of 31–88 years). There were 60 patients with stage I, 56 with stage II, 38 with stage III, and 41 with stage IV oral tumors. Twenty-seven tumors were well-differentiated, 161 were moderately differentiated, and only 1 was poorly differentiated. In this study, we analyzed low T-stage (T1, T2 and T3) oral cancer patients; there were 80 cases with T1 status, 93 with T2 status, and 22 with T3 status. The mean of overall survival was 4.1 years and the median survival time was 3.9 years. The overall survival time ranged from 0.1 to 9.6 years. Adjuvant therapy was administered according to individual considerations.

### 2.2. The Correlation between Cytoplasmic CK1ɛ and CK1δ Expressions in Oral Cancers and Various Clinicopathologic Characteristics

**Figure 1.**
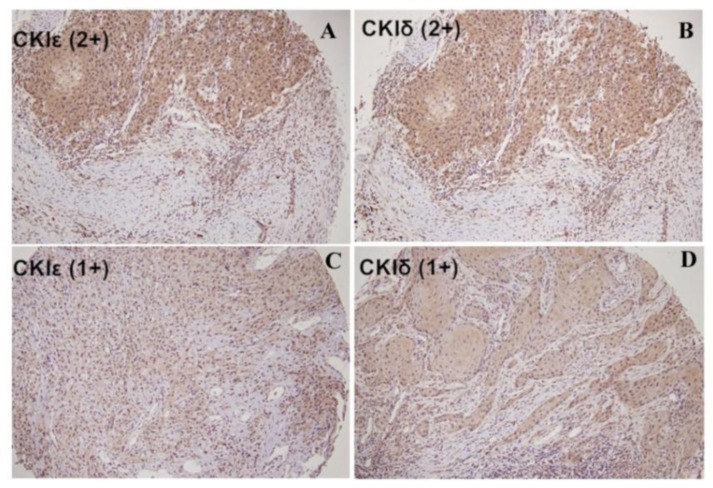

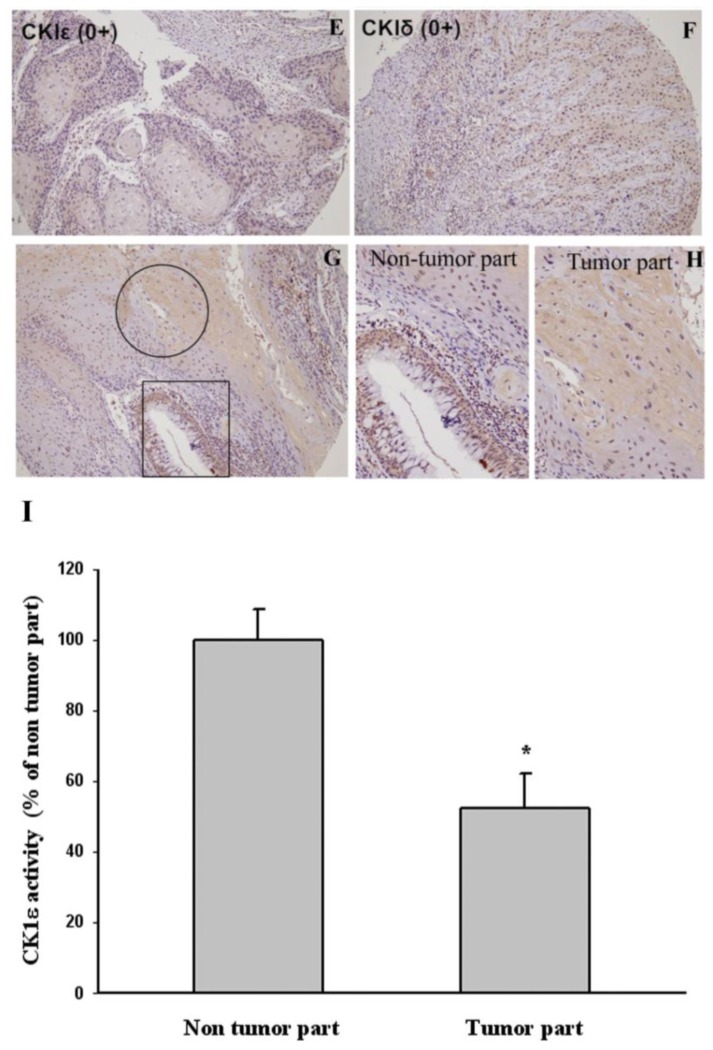
CK1ɛ and CK1δ immunoreactivity and CK1ɛ kinase activity in non-tumor oral squamous mucosa and SCC. (**A**) Strong cytoplasmic CK1ɛ expression in SCC (score 2+); (**B**) Strong cytoplasmic CK1δ expression in SCC (score 2+); (**C**) Weak cytoplasmic CK1ɛ expression in SCC (score 1+); (**D**) Weak cytoplasmic CK1δ expression in SCC (score 1+); (**E**) Negative CK1ɛ expression in SCC (score 0); (**F**) Negative CK1δ expression in SCC (score 0); (**G**,**H**) Strong cytoplasmic CK1ɛ expression in the non-tumor part than that of tumor part. Circle indicated the tumor part. Frame indicated the non-tumor part; and (**I**) Lower CK1ɛ kinase activity in the tumor part then that of non-tumor part. * Significant differences from control values with *p* < 0.05.

Figure 1A–F shows the different CK1ɛ and CK1δ cytoplasmic expression in oral cancer obtained by immunohistochemical analysis. In this study, 35 cases showed a score of 2+ (17.9%), 98 cases received a score of 1+ (50.3%), and 62 cases received a score of 0+ (31.1%) in the CK1ɛ expression. Figure 1G,H shows a strong CK1ɛ expression in non-tumor part by immunohistochemical analysis. We also examined the CK1ɛ kinase activity in the non-tumor part and of tumor part by CK1ɛ kinase activity assay (Figure 1I). The levels of CK1ɛ kinase activity in the tumor part were significantly lower than that in non-tumor parts (*p* < 0.05).

**Figure 2.**
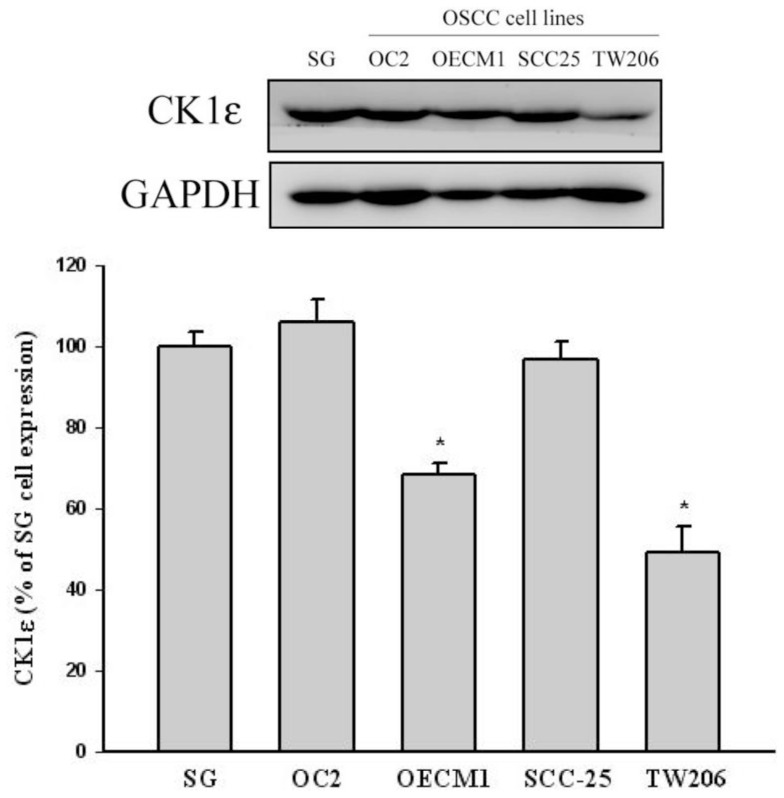
CK1ɛ expression in normal epithelial cell (SG cell) and four different OSCC cells lines (OC2, OECM1, SCC25 and TW206). Western blot analysis, indicating the high expression of CK1ɛ in normal epithelial cells and the low expression in OECM1 and TW206 oral cancer cells. * Significant differences from control values with *p* < 0.05.

We divided the CK1ɛ and CK1δ immunohistological stains into positive (1+/2+) and negative (0) stain subgroups. The correlation between the expression level of CK1ɛ and clinical parameters is summarized in Table 2. The chi-square analyses for the clinicopathologic characteristics of 195 patients with OSCC in relation to cytoplasmic CK1ɛ expression showed that a negative CK1ɛ expression was considerably correlated with a short four-year survival (*p* = 0.002). However, no significant association of CK1δ expression was achieved with gender, age and the clinical parameters. Furthermore, we also test CK1ɛ expression in normal epithelial cells and four different OSCC cells lines. Western blot analysis indicated CK1ɛ expression in all oral cancer cells as well as normal squamous epithelial cells (Figure 2). The expression of CK1ɛ in normal epithelial cells (SG cells) was higher than that in OECM1 and TW206 oral cancer cells (Figure 2).

### 2.3. Correlations between Cytoplasmic CK1ɛ Expression and Patient Survival

**Figure 3.**
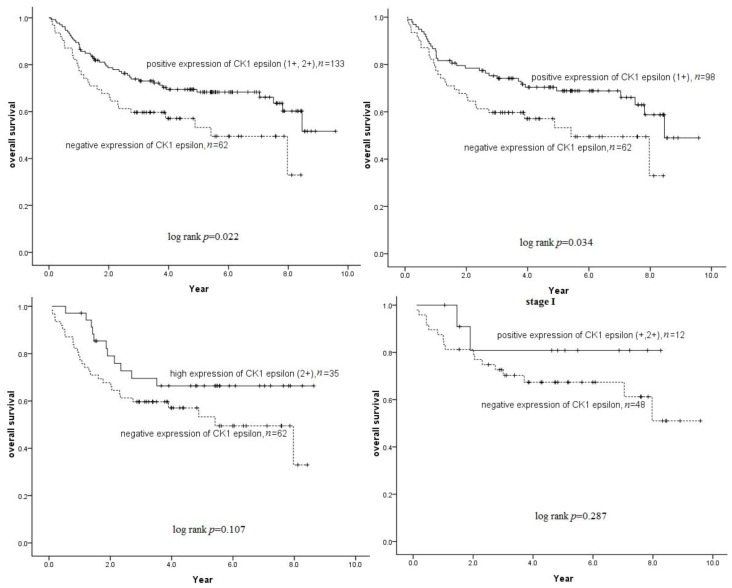

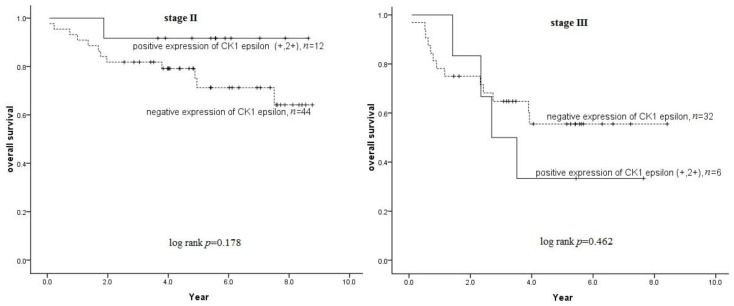
Kaplan-Meier survival curves for oral SCC patients who were classified with either negative or positive cytoplasmic CK1ɛ expression. Loss of CK1ɛ expression was strongly associated (log-rank, *p* = 0.022) with patient survival. However, no significant association was found in the stage I, stage II and stage III subgroups.

In our study, the patients were subdivided into two groups (the negative cytoplasmic CK1ɛ expression group and the positive cytoplasmic CK1ɛ expression group) because cytoplasmic CK1ɛ expression was considerably correlated with patient survival. Survival analysis demonstrated that the probability of survival was considerably lower (*p* = 0.022) in the patients with negative cytoplasmic CK1ɛ expression (Figure 3) than in those who had positive ones. In the subgroup, there are statistical significant association in the negative cytoplasmic CK1ɛ expression group and CK1ɛ expression (score 1+) group. However, no significant association was found in the stage I, stage II and stage III subgroup (Figure 3). Cox proportional regression analysis was used to assess the effect of negative cytoplasmic CK1ɛ expression on overall survival (OS) independently of other clinical variables. In the univariate Cox regression analysis, the results showed that negative cytoplasmic CK1ɛ expression, when adjusted for grade, tumor stage, and lymph node metastasis, retained a statistically significant association with overall survival (*p* = 0.024, hazard ratio (*HR*) = 1.7). The results of the survival analysis showed that the median survival rate in negative cytoplasmic CK1ɛ expression cases was 39.6 months, whereas in positive cytoplasmic CK1ɛ expression cases, it was 57.6 months. The mean survival rate was 40.4 and 54.0 months in negative and positive cytoplasmic CK1ɛ expression cases, respectively. However, CK1δ expression, when adjusted for grade, tumor stage, and lymph node metastasis, had no statistically significant association with overall survival (*p* = 0.475).

In addition, the univariate Cox regression analysis, tumor grade (*p* = 0.009, *HR* = 6.5), disease stage (*p* <0.001, *HR* = 2.3) and lymph node metastasis status (*p* < 0.001, *HR* = 2.8) were independent predictors of poor survival (Table 3). In the multivariate Cox regression analysis (Table 4), tumor grade (*p* = 0.012, *HR* = 6.1) and lymph node metastasis status (*p* = 0.002, *HR* = 2.5) were independent predictors of poor survival. Negative cytoplasmic CK1ɛ expression might be an independent prognostic factor (*p* = 0.052, *HR* = 1.9) for oral cancer patients. These findings suggest that negative cytoplasmic CK1ɛ expression has a major impact on the overall survival of oral cancer patients.

In Taiwan, one of the most prominent cancers affecting people is OSCC. The incidence of this disease in the younger male population has markedly increased during recent years [5]. The prognosis for patients with OSCC remains poor, especially if the disease is not diagnosed at early stages [21,22]. Treatment modalities for oral cancer usually involve surgery and radiation, with or without chemotherapy. Therefore, discovering a method for targeting agents that cause molecular or cellular changes that are specific to oral cancer may have therapeutic potential.

Several studies have demonstrated that CK1ɛ is associated with various cancers and have indicated that CK1ɛ plays a role in carcinogenesis [15,23–30]; contrarily, Fuja *et al.* [22] and Hsu *et al.* [23] reported that CK1ɛ could be a tumor suppressor. Even in the same cancer, CK1ɛ seems to display different functions; it could be an oncoprotein or a tumor suppressor [23,30,31]. In this study, our data showed that there was higher CK1ɛ expression in normal oral squamous mucosa than in tumors. We also discovered that a loss of cytoplasmic expression of CK1ɛ in oral cancer patients is significantly associated with poor overall survival. In the univariate Cox regression analysis, the results showed that the median survival rate in negative cytoplasmic CK1ɛ expression cases was 39.6 months, whereas in positive cytoplasmic CK1ɛ expression cases, it was 57.6 months. The mean survival rate was 40.4 and 54.0 months in negative and positive cytoplasmic CK1ɛ expression cases, respectively. These results indicated that CK1ɛ might be a tumor suppressor in OSCC. Our data is similar to those of Fuja *et al.* [22] and Hsu *et al.* [23]. Fuja and coworkers revealed that CK1ɛ was reduced in poorly differentiated tumors and CK1ɛ showed over-expression in mammary ductal cell carcinoma *in situ* as observed by using an immunohistochemical study [22]. The expression of CK1ɛ showed considerable down-regulation in the cancer tissues (*p* < 0.005) [22]. Hsu *et al.* demonstrated that CK1ɛ expression showed major down-regulation in the cancer tissue in head and neck squamous cell carcinoma by using a real-time quantitative RT-PCR analysis [23]. These studies did not correlate CK1ɛ expression with the patient’s clinical parameters or the patient’s survival statement [24]. However, our data is different from that of Brockschmidt *et al.* [13] because they indicated that there is a high expression of CK1ɛ in cases of ductal adenocarcinoma of the pancreas and that CK1ɛ contributes to aggressive tumor growth. Rodriguez *et al.* [17] also showed that CK1ɛ is considerably over-expressed in ovarian cancer tissues and is associated with poor survival. The differences between our observations and those of Brockschmidt *et al.* [13] and Rodriguez *et al.* [18] could be due to variations in different cancer types. The tumor cell type in their investigations was adenocarcinoma, whereas ours was squamous cell carcinoma. Moreover, we used 195 cancer tissues to conduct our analysis, whereas Brockschmidt *et al.* [13] experimented with only 27 cases of ductal adenocarcinoma of the pancreas, and Rodriguez *et al.* [18] studied only 76 ovarian tumors. The number of specimens we used in our research is greater than that of the other studies; therefore, we believe that this could be a major reason leading to our different results.

It has been suggested that CK1ɛ represents a potential therapeutic target in many types of human cancers. The inhibition of CK1ɛ by IC261 could effectively resensitize cells to apoptosis *in vitro* and reduce tumor growth [15,19,25]. Rodriguez *et al.* [18] also demonstrated that the inhibition of CK1ɛ resulted in decreased cell growth rates and tumor burden, and acted as a potent sensitizer to chemotherapeutic agents. Foldynová-Trantírková *et al.* [30] discovered that the CK1ɛ mutations in breast cancer suppresses Wnt/β-catenin and promotes Wnt/Rac-1-mediated and NFAT-mediated pathways. They indicated that CK1ɛ has the potential to play a role as a tumor suppressor in breast cancer through its negative effect on the Wnt/Rac1/JNK and NFAT pathways.

## 3. Experimental Section

### 3.1. Patients and Samples

In this study, we collected 195 OSCC samples from patients who underwent treatment at Changhua Christian Hospital, Changhua, Taiwan between January 2000 and December 2006. These samples were used to construct a tissue microarray. All of the patients were staged and grades defined by the 2007 American Joint Committee on Cancer staging system. Histopathological and clinical data including patients’ age and sex, tumor status, lymph node metastasis, distance metastasis, betel nut chewing, alcohol drinking, grade, stage and survival were obtained from the cancer registry of Changhua Christian Hospital, Taiwan. Before commencement of this study, approval was obtained from the Institutional Review Board of Changhua Christian Hospital, and informed written consent to participate in the study was obtained from each person.

### 3.2. Tissue Microarrays (TMAs)

Punch of the tumor specimens and non-tumor specimens from hematoxylin and eosin-stained sections: One tissue cylinders with a diameter of 2 mm were made from each OSCC paraffin block using a fine steel needle to produce the tissue microarrays (TMAs). The punches of the tumor specimens were arrayed into new paraffin blocks. A 4-μm hematoxylin and eosin stained section was reviewed by pathologists to confirm the presence of morphologically representative lesions of the original cancers.

### 3.3. Immunohistochemistry

Paraffin embedded squamous cell carcinoma and paired non-cancerous tissue sections (4-μm) on poly- l-lysine-coated slides were deparaffinized in xylene and rehydrated in alcohol. Endogenous peroxidase activity was blocked with 3% H_2_O_2_. The antigen was retrieved by heating at 100 °C for 20 min in 10 mM citrate buffer (pH 6.0). After antigen retrieval, slides were incubated with an affinity-purified goat polyclonal anti-CK1ɛ (C-20) raised against a COOH-terminal peptide of CK1ɛ (Santa Cruz Biotechnology, Santa Cruz, CA, USA) or anti-CK1δ (Abcam; ab10877) for 30 min at room temperature, and washed three times with phosphate buffered saline (PBS). Slides were incubated with an horseradish peroxidase (HRP)/Fab polymer conjugate for another 30 min. The sites of peroxidase activity were visualized using 3,3′-diamino-benzidine tetrahydrochloride as a substrate. Gill Hematoxylin Solution II (MERCK, Darmstadt, Germany) was utilized as the counterstain. Negative controls had the primary antibody omitted and replaced by PBS. Oral mucosa epithelium with homogeneous CK1ɛ cytoplasmic staining was included as the positive controls. A compararative scale from 0 to 2 was used to score staining intensity: “2+” if the staining intensity in the tumor part matched the staining intensity of the normal oral squamous mucosa. The staining was scored as “1+” if the staining intensity in the tumor part was lower than the staining intensity of the normal oral squamous mucosa. A score of “0” reflected a lack of CK1ɛ immunoreactivity compared with the staining pattern of normal oral squamous mucosa. Negative/loss of expression of CK1ɛ was defined as “0”; “1+ and 2+” as positive expression. All immunohistochemical staining cases were examined by two pathologists (Chih-Jung Chen and Kun-Tu Yeh, Department of Surgical Pathology, Changhua Christian Hospital, Changhua, Taiwan), and a final agreement was obtained for each score at a discussion microscope.

### 3.4. Cell and Cell Culture

SG and OC2 cells were cultured in Dulbecco’s modified Eagle’s medium (Life Technologies, Grand Island, NY, USA). OECM1 cells were cultured in RPMI medium. SCC25 and TW206 cells were cultured in Dulbecco’s modified Eagle’s medium supplemented with an equal volume of a nutrient mixture, F-12 Ham’s medium (Life Technologies, Grand Island, NY, USA). All cell cultures were maintained at 37 °C in a humidified atmosphere of 5% CO_2_.

### 3.5. Western Blot Analysis

Cellular lysates were prepared by suspending 2 × 10^6^/10 cm dish in 200 μL of RIPA buffer containing protease inhibitors cocktail. Cell lysates were subjected to a centrifugation of 10,000 rpm for 10 min at 4 °C, and the insoluble pellet was discarded. The 20 μg samples of total cell lysates were separated by SDS-PAGE on 10% polyacrylamide gels and transferred onto a nitrocellulose membrane using the Mini-Protean Tetra Electrophoresis System as described previously [31]. The blot was subsequently incubated with 5% non-fat milk in Tris-buffered saline (20 mM Tris, 137 mM NaCl, pH 7.6) for 1 h to block non-specific binding and then overnight with polyclonal antibodies against CK1ɛ were then incubated with a horseradish peroxidase goat anti-rabbit IgG for 1 h. Afterwards, signal was detected by using enhanced chemiluminescence (ECL) commercial kit (Amersham Biosciences, Piscataway, NJ, USA).

### 3.6. CK1ɛ Kinase Activity

Surgical specimens of human oral cancer and the corresponding paired adjacent normal tissue were obtained right after the surgery, fresh tissue was snap-frozen in liquid nitrogen and stored at −80 °C until processing to determinate CK1ɛ kinase activity by ADP-Glo™ Kinase Assay (Promega, Madison, WI, USA). Briefly, CK1ɛ kinase reactions were carried out for 60 min at room temperature in a 50 μL volume of CK1ɛ kinase-assay buffer (40 mM Tris, pH 7.5; 20 mM MgCl_2_; 0.1 mg/mL BSA; 50 μM DTT) supplemented with dilute enzyme, substrate, ATP, inhibitors and ADP-Glo™ reagent buffer. After incubation at room temperature for 40 min, 10 μL of Kinase detection reagent was added. After 30 min of incubation, luminescence activities were assayed; data are shown as relative light units (RLU) that directly correlate to the amount of ADP produced.

### 3.7. Statistical Analysis

CK1ɛ expression patterns and correlations of CK1ɛ and clinicopathologic parameters of OSCC were examined by chi-squared test. The distribution of overall survival was estimated using a Kaplan-Meier plot and the log-rank test. Overall survival (OS) was defined as the time between the date of diagnosis and the date of death. Independent prognostic factors were analyzed by the Cox proportional hazards regression model (SPSS, Version 17.0; SPSS, Inc., Chicago, IL, USA). The analyses were performed using the Statistical Package for Social Sciences, Version 17.0 (SPSS, Version 17.0; SPSS, Inc., Chicago, IL, USA), and *p* value of less than 0.05 (2-tailed test) was considered to indicate statistical significance.

## 4. Conclusions

In conclusion, our experimental results demonstrated that a loss of cytoplasmic CK1ɛ expression correlates with poor survival rates in oral cancer patients. Our findings suggest that CK1ɛ could not only function as a prognostic predictor for oral cancer, but could also act as a tumor suppressor. Since current viable treatment options are limited for OSCC, continued studies must be conducted to further explore this area and to determine whether CK1ɛ can be used as a pharmacological target for treatments.

## Figures and Tables

**Table 1.** Patient characteristics.

Variable		Oral squamous cell carcinoma	

		*n* = 195	%
Gender	Female	36	18.5
	Male	159	81.5

CK1ɛ expression	0	62	31.8
	1+	98	50.3
	2+	35	17.9

Smoke or Betel nuts	No	71	36.4
	Yes	89	45.6
	Unknown	35	17.9

Stage	I	60	30.8
	II	56	28.7
	III	38	19.5
	IV	41	21.0

Lymph Node Metastasis	No	133	68.2
	Yes	62	31.8

Grade	Well	27	13.8
	Moderate	161	82.6
	Poor	1	3.6

Tumor status	T1	80	41.0
	T2	93	47.7
	T3	22	11.3

Age	Mean	55.9 years	
	Median	55.0 years	
	Range	31−88 years	

Overall-survival	Mean	4.1 years	
	Median	3.9 years	
	Range	0.1−9.6 years	

Follow up	Mean	5.4 years	
	Median	6.0 years	
	Range	0.1−13.2 years	

**Table 2.** Correlation of CK1ɛ expression with clinicopathologic indicators of oral cancer.

Characteristics	Cytoplasmic staining of CK1ɛ (*n* = 195)		*p* value	Cytoplasmic staining of CK1δ (*n* = 173)		*p* value
	
	Negative (%)	Positive (%)		Negative (%)	Positive (%)	
Gender						
F	11 (17.7)	25 (18.8)	0.860	8 (6.8)	3 (5.4)	1.000 ^a^
M	51 (82.3)	108 (81.2)		109 (93.2)	53 (94.6)	

Age						
≤55 years	33 (53.2)	69 (51.9)	0.861	58 (49.6)	30 (53.6)	0.623
>55 years	29 (46.8)	64 (48.1)		59 (50.4)	26 (46.4)	

Grade						
well	10 (16.1)	17 (12.8)	0.529	17 (14.5)	8 (14.3)	0.966
moderate, poor	52 (83.9)	116 (87.2)		100 (85.5)	48 (85.7)	

T status						
T1	29 (46.8)	51 (38.3)	0.211	46 (39.3)	21 (37.5)	0.586
T2	24 (38.7)	69 (51.9)		55 (47.0)	30 (53.6)	
T3	9 (14.5)	13 (9.8)		16 (13.7)	5 (8.9)	

Lymph Node Metastasis						
no	38 (61.3)	95 (71.4)	0.157	75 (64.1)	41 (73.2)	0.233
yes	24 (38.7)	38 (28.6)		42 (35.9)	15 (26.8)	

Distance Metastasis						
no	61 (98.4)	133 (100)	0.413 ^a^	116 (99.1)	56 (100)	1.000 ^a^
yes	1 (1.6)	0 (0)		1 (0.9)	0 (0)	

Stage						
I	19 (30.6)	41 (30.8)	0.079	33 (28.2)	17 (30.4)	0.365
II	12 (19.4)	44 (33.1)		36 (30.8)	20 (35.7)	
III	12 (19.4)	26 (19.6)		20 (17.1)	12 (21.4)	
IV	19 (30.6)	22 (16.5)		28 (23.9)	7 (12.5)	

Survival						
≤4 years	42 (67.7)	58 (43.6)	0.002	45 (38.5)	17 (30.4)	0.298
>4 years	20 (32.3)	75 (56.4)		72 (61.5)	39 (69.6)	

Smoking or Betel Nuts						
no	22 (44)	49 (44.5)	0.949	36 (40.4)	18 (46.2)	0.548
yes	28 (56)	61 (55.5)		53 (59.6)	21 (53.8)	

*p*-value by Fisher’s Exact Test ^a^ or chi-square Test.

**Table 3.** Univariate analysis (Cox regression) of several clinicopathologic indicators of oral cancer.

Variable	Hazard ratio	95% CI	*p* value
Expression of CK1ɛ (*n* = 195)			
negative	1.7	0.361–0.930	0.024
positive	1.0		

Grade			
well	1.0	1.601–26.628	0.009
moderate/poor	6.5		

Stage			
I + II	1.0	1.470–3.717	<0.001
III + IV	2.3		

Lymph Node Metastasis			
no	1.0	1.740–4.379	<0.001
yes	2.8		

Expression of CK1δ (*n* = 173)			
negative	1.2	0.462–1.432	0.475
positive	1.0		
Grade			
well	1.0	1.328–13.458	0.015
moderate/poor	4.2		

Stage			
I + II	1.0	1.560–4.101	<0.001
III + IV	2.5		

Lymph Node Metastasis			
no	1.0	1.549–4.060	<0.001
yes	2.5		

**Table 4.** Multivariate analysis (Cox regression) of several clinicopathologic indicators of oral cancer.

Variable	Hazard ratio	95% CI	*p* value
Expression of CK1ɛ (*n* = 195)			
negative	1.6	0.382–1.004	0.052
positive	1.0		

Grade			
well	1.0	1.490–25.080	0.012
moderate/poor	6.1		

Stage			
I + II	1.1	0.364–2.424	0.897
III + IV	1.0		

Lymph Node Metastasis			
no	1.0	1.390–4.665	0.002
yes	2.5		

Expression of CK1δ (*n* = 173)			
negative	1.1	0.495–1.540	0.638
positive	1.0		

Grade			
well	1.0	1.180–19.992	0.029
moderate/poor	4.9		

Stage			
I + II	1.0	0.828–4.106	0.134
III + IV	1.8		

Lymph Node Metastasis			
no	1.0	0.771–3.771	0.188
yes	1.7		
